# Roles of Ceramides in Non-Alcoholic Fatty Liver Disease

**DOI:** 10.3390/jcm10040792

**Published:** 2021-02-16

**Authors:** Eric Hajduch, Floriane Lachkar, Pascal Ferré, Fabienne Foufelle

**Affiliations:** 1 Centre de Recherche des Cordeliers, INSERM, Sorbonne Université, Université de Paris, 75006 Paris, France; eric.hajduch@crc.jussieu.fr (E.H.); floriane.lachkar@sorbonne-universite.fr (F.L.); pascal.ferre@crc.jussieu.fr (P.F.); 2 Institute of Cardiometabolism and Nutrition (ICAN), Hôpital Pitié-Salpêtrière, AP-HP, 75013 Paris, France

**Keywords:** ceramide, liver, NAFLD, NASH, sphingolipids, steatosis

## Abstract

Non-alcoholic fatty liver disease is one of the most common chronic liver diseases, ranging from simple steatosis to steatohepatitis, fibrosis, and cirrhosis. Its prevalence is rapidly increasing and presently affects around 25% of the general population of Western countries, due to the obesity epidemic. Liver fat accumulation induces the synthesis of specific lipid species and particularly ceramides, a sphingolipid. In turn, ceramides have deleterious effects on hepatic metabolism, a phenomenon called lipotoxicity. We review here the evidence showing the role of ceramides in non-alcoholic fatty liver disease and the mechanisms underlying their effects.

## 1. Introduction on NAFLD

### 1.1. Steatosis and Non-Alcoholic Steatohepatitis 

Non-alcoholic fatty liver disease (NAFLD), or as recently proposed metabolic associated fatty liver disease [1], encompasses a spectrum of pathologies ranging from benign steatosis characterized by triglyceride (TG) accumulation in hepatocytes to non-alcoholic steatohepatitis (NASH) that can progress to fibrosis, cirrhosis, and hepatocellular carcinoma (HCC)**.** NAFLD is also associated with extra-hepatic complications such as cardiovascular diseases that are the leading cause of mortality in NASH and fibrotic patients. In the coming years, NAFLD is expected to become one of the leading causes of liver transplantation both for end-stage liver disease and HCC. Although NAFLD etiology is heterogeneous and complex, the basic mechanisms explaining the initial fat storage in hepatocytes could be an increased inflow of fatty acids coming from the diet or from adipose tissue into the liver, increased fatty acid synthesis from carbohydrates (de novo hepatic lipogenesis), a decreased fatty acid oxidation, and a decreased export of TG in the form of very-low-density lipoproteins (VLDL). 

NASH represents an advanced form of fatty liver disease in which lipid accumulation is associated with cell injury and lobular inflammation and occasionally with fibrosis. The progression towards NASH is currently explained by a “multiple parallel-hit” hypothesis since simple liver fat accumulation is not sufficient to cause inflammation and fibrosis. Multiple signals originating directly from diseased hepatocytes or from other organs are considered secondary hits. Among them, lipotoxic lipids synthesized from the free fatty acids released by adipose tissue or synthesized by de novo lipogenesis can induce oxidative stress or endoplasmic reticulum (ER) stress, which ultimately results in cell injury. Cell injury induces the recruitment and activation of inflammatory cells. The combination of inflammation and tissue damage triggers hepatic stellate cells (HSC) activation and collagen deposition. Here, we will review how ceramides participate in the onset of steatosis and its transition to NASH by promoting ER stress and hepatocyte injury. We will also see how ceramides participate in inflammation and fibrosis in other liver cells.

### 1.2. NAFLD, Obesity and Diabetes

Obesity and Type 2 diabetes (T2D) are two conditions leading to an increased prevalence of NAFLD. It affects around 25% of the general population of western countries but over 60% in obese subjects and can reach 90% in Type 2 diabetic patients [2]. A key factor common to these two syndromes is insulin resistance. Insulin resistance is defined as a decreased effect of the hormone on its targets for a similar concentration of plasma insulin. Insulin resistance is the consequence of an increased fat availability originating initially from a disequilibrium between intake and expenses. If the storage capacity of adipose tissue is exceeded, fat overflow in various organs (muscles, liver, pancreatic ß-cells) leads to the synthesis of specific lipids, such as sphingolipids (SL) which interfere with insulin signaling/secretion. This important concept of lipotoxicity was initially proposed by RH Unger [3]. The hypertrophy of adipocytes during obesity induces the secretion of pro-inflammatory cytokines such as tumor necrosis factor α (TNFα) or interleukin 6 (IL6) but also the recruitment of immune cells which participate locally in adipose tissue inflammation, leading to adipose tissue insulin resistance. One major action of insulin in adipose tissue is the inhibition of lipolysis, i.e., the release of fatty acids from adipocytes into the bloodstream. Insulin resistance will thus accelerate the overflow of fatty acids to various organs including the liver. In muscles, a major site of glucose utilization, insulin resistance reduces glucose uptake and this will induce a compensatory increase of insulin secretion and hyperinsulinemia. In turn, this hyperinsulinemia will activate de novo hepatic lipogenesis which in contrast to other hepatic insulin-dependent pathways such as glucose production through gluconeogenesis, seems to remain insulin sensitive. This paradox was explained by Brown and Goldstein [4] since the mammalian target of rapamycin complex 1 (mTORC1), a downstream effector of insulin action is essential for lipogenesis stimulation but not for gluconeogenesis inhibition. We will see later other explanations for this paradox that do not involve insulin. The activated lipogenesis reinforces hepatic fatty acid availability for TG synthesis and storage. Another consequence of activated de novo lipogenesis is the inhibition of fatty acid oxidation since an intermediate of lipogenesis, malonyl-CoA inhibits the entry of fatty acids into mitochondria, a prerequisite for their oxidation, thus reducing fat clearance. Thus, insulin resistance by increasing fatty acid availability for the liver (adipose tissue lipolysis and hepatic lipogenesis) is an important factor for NAFLD development.

Conversely, an important aspect of hepatic steatosis is the generation of specific lipid species (see below) which will favor hepatic insulin resistance leading to an unabated glucose production by the gluconeogenic pathway, glucose intolerance, and favoring the onset of T2D. Indeed, in prospective studies, the presence of NAFLD at inclusion was associated with an odds ratio of 2.4 for developing diabetes [5].

Thus, hepatic steatosis in NAFLD is a consequence of obesity but will, in a vicious circle, aggravate the syndrome by exacerbating insulin resistance.

## 2. Overview of Sphingolipid and Ceramide Metabolism

**Figure 1:**
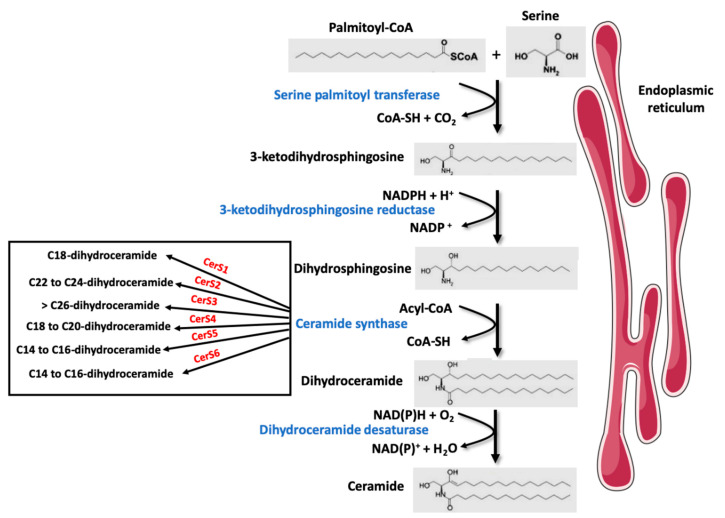
Ceramide de novo synthesis pathway.

SL are a major class of eukaryotic lipids. When they were discovered by Thudichum in the 1850s, they were named after the Greek mythological creature, the sphinx, due originally to their enigmatic function [6]. SL play important structural roles in cell membranes (e.g., Sphingomyelin (SM), gangliosides) but they are also able to modulate multiple cell functions, such as cell death (e.g., ceramide and sphingosine), cell survival (e.g., sphingosine-1-phosphate (S1P)), cell proliferation (e.g., ceramides, ceramide-1-phosphate), senescence (e.g., ceramide), insulin response, and inflammation (e.g., ceramides, S1P) [7,8,9,10,11]. SL are a class of lipids defined by their sphingoid backbone consisting of 18 carbons comprising amine and alcohol groups. In vertebrates, this base can be sphingosine or dihydrosphingosine [12]. Ceramides are precursors for the biosynthesis of SL such as SM or glycoceramide, themselves precursors for other SL. Ceramides can be synthesized via the de novo synthesis pathway but they can also be produced through the sphingomyelinase pathway and/or the so-called “recycling” pathway. The de novo ceramide synthesis pathway occurs in the cytosolic leaflet of the ER, where four enzymes (or enzyme families) generate ceramides from a non-SL precursor (palmitate) and the amino acid serine, followed by the addition of acyl-CoA of different chain lengths (Figure 1). This pathway is predominant in the lipid excess context encountered during obesity. The first step of this synthesis involves serine palmitoyl transferases (SPT) which condenses a serine with a palmitoyl-CoA to produce 3-ketodihydrosphingosine [12]. The second step is carried out by 3-ketodihydrosphingosine reductase that reduces 3-keto-dihydrosphingosine in an NADPH-dependent manner to form dihydrosphingosine (also called sphinganine) [12]. The third step involves a family of enzymes called ceramide synthases (CerS). These enzymes catalyze the addition of fatty acids of variable chain length and unsaturation on dihydrosphingosine, resulting in the formation of dihydroceramides (DhCer) [13]. In mammals, six CerS isoforms, called CerS 1 to 6, are expressed. Each isoform displays specificities for the chain length of the added acyl-CoA, although redundancies exist between the different isoforms [13]. The existence of so many isoforms dedicated to carrying out a single enzymatic reaction suggests that depending on their chain length, ceramides and their derivatives possess different properties. The final reaction for ceramide production is catalyzed by DhCer desaturase-1 (DES1), responsible for the desaturation of the dihydrosphingosine backbone into a sphingosine backbone to ultimately give a ceramide molecule [13]. 

Once synthesized, ceramides are then immediately transferred by transporters into the Golgi apparatus [12,14,15] where they serve as precursors for the synthesis of complex SL such as SM and glucosylceramides (precursors of gangliosides) [13]. While SM are major structural components of cell membranes, glucosylceramides play essential roles in cell growth, differentiation, and receptor-mediated signal transduction [16]. 

In addition to de novo synthesis, ceramides can also be generated in the cell through hydrolysis of SM. Hydrolysis of SM is catalyzed by sphingomyelinases (SMase) that catalyzes the cleavage of the phosphocholine head group of SM to generate ceramides. 

Finally, other complex SL can also be “salvaged” into ceramide. This process takes place in the late endosomes and lysosomes and involves several enzymes such as cerebrosidases, ceramidases, and CerS [17]. In physiological conditions, this SL turnover pathway has been estimated to contribute from 50% to 90% of ceramide biosynthesis [17]. 

In this review, we will focus on the role of ceramides since among the sphingolipid species, it is the most frequently found to be involved in the different steps of NAFLD progression.

## 3. Role of Sphingolipids in Hepatic Steatosis

### 3.1. Liver Sphingolipids Concentrations are Altered in NAFLD 

Liver sphingolipid species have been analyzed in numerous rodent models of NAFLD [18,19,20,21,22,23,24]. An increase in total liver ceramide concentration was found in many but not all studies [25,26,27]. These divergent data could be explained by two reasons. First, during an increased fatty acid availability, ceramides do not accumulate in the liver as much as observed in other peripheral tissues [28]. Indeed, it seems that the liver can detect precisely intracellular ceramide concentration, and prevents its build-up by increasing its secretion into the circulation mainly within VLDL lipoproteins later transformed into LDL, probably to protect the liver from ceramide accumulation [28]. Thus, the liver is considered a primary source of circulating ceramides. Second, a recent study showed that hepatic lipotoxicity induces an enhanced conversion of biologically active ceramides into inert acylceramides and their accumulation into lipid droplets to form an “intracellular inactive ceramide storage pool”, thus preventing ceramides to accumulate in liver membranes [29]. Nevertheless, in the studies which have measured the different ceramide species, a common trend emerges: steatosis is concomitant with an increase in ceramides 14:0, 16:0, 18:0, 20:0 and a decrease in ceramides with a longer acyl-chain, such as ceramide 24:0. Most studies have shown that the increase in ceramides during hepatic steatosis is linked to an enhanced SL de novo synthesis with an increase in the expression of enzymes, SPT, CerS 1, 2, 4, 6 [19,23,24,30]. However, changes in specific ceramides do not always correlate with the corresponding CerS expression/activity [31]. In addition to CerS, acid SMase which generates ceramide from SM has also been involved in the increased ceramide concentrations in NAFLD [32].

Interestingly, lipodystrophic syndromes are also concomitant with hepatic steatosis due to a reduced capacity of adipose tissue fat storage, with insulin resistance, and a susceptibility to develop type 2 diabetes [33]. In a rodent model of lipodystrophy generated by the knock-out of 1-acylglycerol-3-phosphate O-acyltransferase 2 (Agpat2−/−), a general increase in the sphingolipid pathway was observed with robust increases in C16:0 and C18:0 ceramides in the liver and the plasma linked to an increased expression of SPT and CerS5 and CerS6 [34].

Studies have analyzed the changes in hepatic ceramide concentrations with the progression of steatosis to NASH in rodents [35,36]. Ceramide accumulation and increased expression of enzymes involved in their synthesis have been described in mouse models of NASH even in the absence of obesity (methionine choline-deficient diet) [37,38]. In rodents, pharmacological studies using myriocin, an inhibitor of STP, showed that diminution of hepatic ceramide content can prevent NASH features [39].

The studies in humans are less numerous. One compared in liver and plasma a number of lipid species in order to discriminate control subjects from patients with steatosis, NASH, and cirrhosis [40]. Whereas ceramide concentrations were not different among the four groups in the liver, in the plasma, ceramides 18:0, 20:0, 22:0, 24:0, 24:1, and DhCer 18:0 and 24:1 were similar in steatotic patients and control subjects but higher in the NASH group and lower in the cirrhosis group. Luukkonen et al. [41] studied patients undergoing bariatric surgery. They were classified as having a low or a high insulin resistance calculated from the HOMA index [42] and were also different in terms of hepatic steatosis with respectively a low and a high liver fat content. Ceramide (16:0, 18:0, 22:0, 24:1) concentrations increase in the liver of patients with a high-fat content and insulin resistance. Interestingly, liver DhCer (16:0, 18:0, 23:0, 24:1) also increases whereas markers of SM hydrolysis and the ceramide salvage pathway do not. This suggests that the increase in hepatic ceramides is mainly due to the activity of the de novo synthesis pathway. In keeping with this, hepatic palmitate concentrations were increased in the liver of the high-fat insulin-resistant group. 

Apostolopoulou et al. [43] have studied lean control subjects and insulin-resistant obese patients with or without NAFL and NASH. Hepatic total ceramide and DhCer 16:0, 22:0, and 24:1 were elevated in NASH and serum total ceramides and DhCer 22:0 and 24:1 correlated negatively with whole-body insulin sensitivity. It was also shown recently in a cohort of 73 patients that the expression of several enzymes of SL de novo synthesis increased with the severity of NAFLD [44]. Weight loss in NASH patients was concomitant with a decrease in hepatic expression of ceramide synthesizing genes and in circulating ceramide [45].

Thus, in humans, ceramides increase in the liver of patients with NAFLD, but this seems even more marked with NASH than with simple steatosis. The de novo pathway of ceramide synthesis could be responsible for this increase. However, the number of studies and patients is presently rather low and these results should be confirmed in further studies. 

Interesting observations were made on DhCer, the precursors of ceramides. Plasma DhCer concentrations were elevated in humans in several conditions associated with NAFLD. Plasma 18:0, 20:0, 22:0, 24:1 DhCer but not plasma ceramides were associated with waist circumference, a marker of central obesity [46]. Plasma levels of C18:0, C20:0, and C22:0 ceramides, as well as C24:1 DhCer, are elevated in obese female children and adolescents with T2D and correlate with insulin resistance and plasma TG [47]. In type 2 diabetic patients, plasma DhCer 18:0, 20:0, 22:0, 23:0, and 24:0 but not plasma ceramides were increased when compared to control individuals [48]. Wigger et al. [49] reported that in humans, plasma DhCer is the single lipid class increased up to nine years before and after diabetes onset. All the metabolic situations described above were concomitant with an increased prevalence of NAFLD and the presence of NAFLD was associated with an increased risk of 2.4–3.5 for developing diabetes [2]. These observations can be explained by the recent finding [44] that plasma 18:0, 22:0, 23:0, 24:0, 24:1 DhCer are associated with NAFLD severity since they are found in plasma TG-rich VLDL which are increased with hepatic steatosis. This suggests a specific role for these SL in the synthesis or secretion of TG-enriched VLDL. 

### 3.2. Liver Ceramide Contribution to NAFLD 

What can be the consequences of the increased synthesis and concentrations of these SL in the liver during NAFLD?

#### 3.2.1. Sphingolipids and Hepatic Insulin Resistance

It is now clear that ceramides play an essential role in the modulation of glucose homeostasis by negatively targeting the insulin signaling pathway in organs such as skeletal muscles, adipose tissue, and the liver.

In the liver, the insulin signaling pathway is initiated by the binding of the hormone to its receptor. Activation of the latter by autophosphorylation of its β subunits leads to phosphorylation of tyrosine residues of intracellular target substrates, in particular insulin receptor substrates (IRS). Phosphatidylinositol 3-kinase (PI3K) is an essential player in the insulin cascade. Indeed, in response to the hormone, PI3K binds to the tyrosine-phosphorylated IRS and is activated. It induces the formation of a specific membrane phosphoinositides (phosphatidylinositol-3,4,5-triphosphate) which triggers the recruitment to the plasma membrane of the protein kinase Akt (also called protein kinase B, PKB), and thus allows its activation after phosphorylation of two key amino acids, Threonine 308 and Serine 473 respectively by the kinases Phosphoinositide-dependent kinase-1 and mTORC2 [50,51]. Once activated, Akt phosphorylation stimulates liver glycogen synthesis, glycolysis, and lipid synthesis and inhibits hepatic glucose production (gluconeogenesis) [50].

Conversely to skeletal muscle and fat where a major involvement of ceramides in the onset of insulin resistance is well established [52,53], their implication in hepatic insulin resistance is rather recent. As described above, several studies showed increased levels of ceramide concentration in livers of obese and insulin-resistant rodent models. 

Although many studies show a positive correlation between hepatic concentrations of ceramides and the onset of hepatic insulin resistance, others suggest that another lipid species, diacylglycerols synthesized during the esterification of fatty acids into TGs are also involved in insulin resistance [54,55]. 

The implication of ceramides in hepatic insulin resistance has been strengthened recently. As mentioned above, C16-ceramide levels were found to be significantly elevated in livers of several mouse strains fed a high-fat diet (HFD) [24,56], and studies demonstrated that this ceramide species played a crucial role in the development of hepatic insulin resistance. One study showed that CerS2 haploinsufficiency in mice decreased very-long-chain (C22/C24/C24:1) ceramides, but led to a concomitant increase of C16-ceramide that conferred a higher predisposition to HFD-induced insulin resistance [18]. Similar results were obtained in CerS2 null mice. These mice exhibited glucose intolerance and both insulin receptor and Akt phosphorylation were abrogated in the liver, but not in adipose tissue or in skeletal muscle [57]. Conversely, overexpression of CerS2 in primary mouse hepatocytes induced a specific accumulation of long-chain ceramides and improved insulin signaling [56].

Since CerS6 mediates C16-ceramides de novo synthesis, liver-specific CerS6 deficient mice under a HFD had lower C16-ceramides and displayed better glucose tolerance and hepatic insulin sensitivity than wild-type littermates on the same diet [58]. These data were confirmed recently by selectively inhibiting CerS6 using antisense oligonucleotides (ASO) in obese insulin-resistant animal models (ob/ob mice and HFD-fed mice). CerS6 ASO-injected mice displayed a 50% reduction in hepatic C16-ceramide content compared to untreated animals. A similar C16-ceramide reduction was observed in the plasma, confirming that circulating plasma C16-ceramides are mostly generated from the liver [24]. CerS6 ASO treatment improves glucose tolerance and insulin sensitivity in both animal models compared to untreated animals [24]. Interestingly, overexpression of CerS6 in isolated primary hepatocytes increases C16-ceramide content while lowering levels of C22/C24 ceramides derived from CerS2 [18]. CerS6 overexpression inhibits insulin-induced Akt phosphorylation and promoted TG accumulation [18].

Another study confirmed the involvement of ceramides in hepatic insulin resistance. Liver-specific deletion of DES1 (the enzyme which produces ceramides from DhCer) prevented insulin resistance in mice caused by leptin deficiency (ob/ob) or HFD, strengthening the importance of ceramide as a regulator of glucose homeostasis [59]. Overall, these data suggest that C16-ceramides are deleterious ceramide species in the liver, while very-long-chain ceramides (C22-24) display protective functions in this tissue [60,61]. Therefore, these ceramide species could be interesting targets to improve insulin sensitivity.

#### 3.2.2. Mechanisms of Ceramide-Induced Hepatic Insulin Resistance

Ceramides target the insulin signaling pathway in insulin-sensitive tissues through the inhibition of Akt. They activate the atypical protein kinase C isoform PKCζ which interacts and phosphorylates Akt on a Thr34/Ser34 residue, inducing the sequestration of Akt into specialized domains of the plasma membrane called caveolae, thus preventing Akt to be recruited to the plasma membrane where it is normally activated in response to insulin [62,63,64,65]. In cells lacking caveolae, ceramides can inhibit Akt through another mechanism involving its dephosphorylation by protein phosphatase 2A (PP2A) [63,66,67]. In the liver, the ceramide mechanism of action on insulin signaling has not been fully elucidated. However, since the density of caveolae in hepatocytes is relatively low compared to muscle cells and adipocytes [68], and the liver is among the tissues with the lowest relative level of expression of Caveolin-1, the structural protein of caveolae in the plasma membrane [68], the ceramide/PP2A axis must prevail in this tissue. 

Another alternative pathway known to modulate insulin signaling has also been found in hepatocytes. This pathway involves the double-stranded RNA-dependent protein kinase (PKR), a protein involved in the initiation of innate immune defenses [69]. In HepG2 liver cells, ceramide-activated PKR interferes with the insulin signaling pathway by phosphorylating IRS1 on a serine residue in an IκB kinase (IKK)/c-Jun N-terminal kinase (JNK)-dependent manner, thus preventing IRS1 association with the PI3K [70]. A similar mechanism exists in muscle cells [71]. 

Thus, ceramides, by inducing hepatic insulin resistance, will favor elevated glucose production, hyperglycemia, and hyperinsulinemia which in turn can promote the de novo synthesis of lipids (see below). However, ceramides can also participate in NAFLD independently of their effect on insulin resistance.

#### 3.2.3. Direct Effect of Sphingolipid Species on Hepatic Lipid Metabolism

NAFLD is concomitant with increased lipid deposition in hepatocytes. Several mechanisms can contribute to this phenomenon: increased fatty acid uptake and endogenous fatty acid production (lipogenesis) and decreased fatty acid export through VLDL, and decreased fatty acid oxidation. 

Fatty acid uptake depends upon the availability of plasma fatty acids and upon the capacity of fatty acid transport. Several studies have shown that plasma free fatty acids and particularly saturated and monounsaturated fatty acids (palmitate, oleate, palmitoleate) are increased in the plasma of subjects with non-alcoholic fatty liver and NASH [72,73,74,75,76]. It fits with the insulin resistance of adipose tissue, favoring lipolysis. 

Lipid uptake in hepatocytes is carried out by a family of fatty acid transport proteins (FATP), FATP2 and 5 being the most highly expressed in the liver, and by CD36 also called fatty acid translocase. They act in synergy with acyl-CoA synthetase to provide fatty acyl-CoA [77]. In obese rodent models, decreasing hepatic ceramide concentrations either by overexpression of acid ceramidase or by the invalidation of DES1 led to a decreased fatty acid uptake through a reduced expression of CD36, FATP2, and 5 and a reduced translocation of CD36 to the plasma membrane [59,78]. The cellular mechanism explaining these effects is reduced activity of PKCζ a known ceramide target. In humans, elevated plasma free fatty acids and increased hepatic uptake can explain the fact that 60% of hepatic TGs are derived from plasma free fatty acids [79]. 

NAFLD is associated with increased plasma TG-enriched VLDL concentrations [76,80,81], suggesting that impaired VLDL secretion is not a primary factor explaining excessive fat storage. In fact, VLDL-TG secretion is less sensitive to insulin inhibition in NAFLD subjects (hepatic insulin resistance) [82], implying that the secretion of larger VLDL particles remains unabated during feeding cycles, thus contributing to dyslipidemia observed in NAFLD subjects. Nevertheless, it must be pointed out that Lytle et al. [83] described an inverse correlation between intrahepatic fat content and VLDL-TG secretion rate. 

Whereas the role of a decreased VLDL-TG secretion in NAFLD is debated, the importance of endogenous free fatty acid synthesis is more consensual. Several studies using stable isotopes have shown that lipogenesis is increased 3 to 5 fold in human NAFLD subjects and can account for up to 25–40% of TG fatty acids [75,76,79,84]. Interestingly, the comprehensive study of Lambert et al. [76] shows that if one considers the various sources of TG produced by the liver, free fatty acids coming from adipose tissue, meals, or lipogenesis, lipogenesis is the only one which is markedly increased in NAFLD patients.

Lipogenesis is the de novo synthesis of fatty acids from carbohydrates. As such it involves the cytosolic glycolytic pathway yielding pyruvate as a provider of carbons. Pyruvate enters into mitochondria and is transformed into acetyl-CoA by pyruvate dehydrogenase. The lipogenic pathway per se starts from acetyl-CoA to yield finally palmitate, a 16-carbon saturated fatty acid that can then be desaturated and elongated. Key lipogenic enzymes include ATP-citrate lyase, acetyl-CoA carboxylase (ACC), fatty acid synthase, and stearoyl-CoA desaturase. Interestingly, the product of the reaction catalyzed by ACC, malonyl-CoA is an inhibitor of carnitine palmitoyltransferase 1, a key step of fatty acid oxidation. It implies that lipogenesis and fatty acid oxidation are theoretically not concomitantly active. Regulation of lipogenesis is strongly dependent upon the nutritional conditions since it can be considered as a way to convert excess carbons from dietary carbohydrates into fat stored in adipose tissue. It is regulated in a complex manner at a post-translational level and at a transcriptional level by glucose itself and by insulin [85]. Key transcriptional factors are SREBP-1c (sterol regulatory element binding protein 1c) activated by insulin and ChREBP (carbohydrate regulatory element binding protein) activated by glucose [85]. They control the expression of all lipogenic genes and specific glycolytic genes, glucokinase and pyruvate kinase. The inactive precursor of SREBP-1c is a membrane-bound protein of the ER and is associated with a protein called SCAP (for sterol cleavage activating protein). The complex is retained in the ER through an association with a membrane protein called Insig-1 (insulin-induced gene). In the presence of insulin, the SREBP-1c/SCAP complex dissociates from Insig-1 and moves to the Golgi where SREBP-1c is cleaved to yield the mature active transcription factor which activates the expression of lipogenic genes and the SREBP-1c gene itself. 

In animal models of hepatic steatosis [86,87] and in livers of humans with NAFLD [88,89,90], the expressions of SREBP-1c and its target genes are increased.

Several studies report that ceramides can activate SREBP-1c expression and cleavage. In CHO cells, ceramide synthesis is associated with transcriptionally active SREBP- 1 and the expression of its target genes [91]. Primary mouse hepatocytes treated with C2-ceramides show an increase in the expression of the SREBP-1c gene and its transcriptionally active nuclear form [92]. C16-ceramide injection in high-fat-fed mice is also concomitant with the expression of SREBP-1c and its target genes [92].

In Hep3B cells, CerS6 overexpression which increases the concentration of C16-ceramides induces the cleavage of SREBP-1c [93]. Finally, the ablation in mice of DES, the enzyme which produces ceramide from DhCer drastically decreases the hepatic expression of SREBP 1c and its target genes [59].

What can be the link between ceramide and SREBP-1c activation, considering that the strong insulin resistance observed in the fatty liver should impair the effect of insulin, the usual inducer of SREBP-1c expression and cleavage? Two non-exclusive explanations have been proposed. The first one involves the PKCζ which is downstream of PI3K activation by insulin and increases the expression of SREBP-1c [94]. As mentioned above, ceramides stimulate PKCζ, implying that despite the insulin resistance, lipogenesis can be activated. However, the mechanism involved in the stimulation of SREBP-1c cleavage and expression by PKCζ is unknown. The second possibility is activation of SREBP-1c cleavage by an ER stress. In the liver, the ER is an organelle in which takes place the folding and post-translational modifications of the membrane and secreted proteins, the synthesis of lipids and cholesterol for membrane formation and for TG storage. ER is also an important place for cell calcium signaling. If the homeostasis of ER is perturbed, a response called unfolded protein response (UPR) is launched aiming at restoring ER homeostasis [95]. Interestingly, ER stress is present in steatotic livers in both animal models and humans [95]. We have shown that an ER stress is able to induce the cleavage of SREBP-1c leading to an enhanced lipogenesis, possibly through a decrease in the Insig-1 protein or through the dissociation of a chaperone protein called binding immunoglobulin protein/glucose regulated protein 78 [86]. Jiang et al. [92] showed that incubation of primary mouse hepatocytes with C2 ceramide increased their TG content, the expression and the mature nuclear form of SREBP-1c, and its target genes. Kim et al. [93] have incubated Hep3B cells with ceramides of different chain lengths in the presence of palmitate. C16–C18 ceramide enhanced the ER stress caused by palmitate, an effect recapitulated by the overexpression of CerS6, the CerS which synthetizes C16-ceramides. CerS6 overexpression decreased the protein Insig-1 leading to increased SREBP-1c cleavage and thus to enhanced lipogenesis. Interestingly, decreasing the ER stress inhibited the effects of CerS6 overexpression on palmitate-induced SREBP-1 cleavage. Thus, a potential mechanism relating ceramides and lipogenesis in steatotic livers is the induction of an ER stress by ceramides leading to Insig-1 down-regulation, SREBP-1c transfer, and cleavage in the Golgi, and activation of lipogenic genes. Although no direct demonstration is available in the human steatotic liver, higher concentrations of ceramide and particularly C16-ceramides, the presence of an ER stress, higher SREBP-1c and lipogenic gene expression, and a stimulated lipogenesis render this mechanism highly plausible. The mechanisms for the induction of an ER stress by ceramides are unknown but could, for instance, involve a direct effect on the physical properties of ER membranes as shown for saturated fatty acids [96] (and see below). 

The last mechanism which can explain hepatic fat accumulation is a decreased free fatty acid oxidation. In addition to the potential inhibitory effect of malonyl-CoA on fatty acyl-CoA entry into mitochondria due to the activated lipogenesis, a direct effect of ceramides on mitochondrial oxidation capacity was described. CerS2 haploinsufficiency in rodents which decreases very-long-chain C22/C24/C24:1 ceramides induces a compensatory increase in C16-ceramide. This is concomitant with a decreased capacity for palmitate oxidation and a decreased activity of complexes II and IV of the electron transport chain [18]. Conversely, deletion of CerS6 which synthetizes C16-ceramides improves palmitate oxidation and mitochondrial respiration [58]. The underlying mechanisms are not entirely solved but can involve an effect of ceramides on mitochondrial fission which leads to a decreased mitochondrial efficiency [97,98].

**Figure 2:**
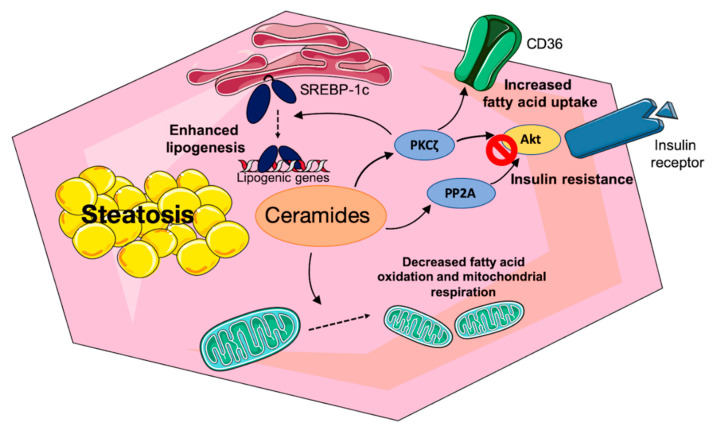
Actions of ceramides on hepatocyte lipid metabolism. Ceramides increase the availability of fatty acids for triglyceride synthesis by promoting the expression and transfer to the plasma membrane of the fatty acid transporter CD36, by inducing the cleavage of the precursor form of the transcription factor SREBP-1c which activates the expression of lipogenic genes, and by reducing fatty acid oxidation in mitochondria. Ceramides also induce hepatic insulin resistance by reducing the insulin-dependent activation of the protein kinase Akt, thus contributing to dysfunctional glucose metabolism.

The various actions of ceramides on lipid metabolism in hepatocytes are summarized in Figure 2.

## 4. Ceramides and NASH

### 4.1. Ceramides and ER Stress 

Activation of ER stress is one important event observed in lipid overwhelmed hepatocytes. It is the consequence of misfolded proteins accumulation in the ER lumen due to an imbalance between the flow of protein synthesis and the ability to fold these proteins. Hepatocytes that are highly enriched in ER are particularly prone to ER stress. As stated above, the cell response to ER stress is the activation of UPR in order to maintain ER homeostasis. The UPR is composed of three signaling branches: inositol requiring enzyme 1 (IRE1), protein kinase RNA-activated-like ER kinase (PERK), and activating transcription factor 6 (ATF6). Whereas mild ER stress is considered to be cytoprotective, prolonged and intense ER stress promotes cell death. Increased expression of UPR markers is observed in the livers of NAFLD rodent models as well as in human livers [99]. Liver fat overload is probably a major factor in the initiation of chronic ER stress. Saturated fatty acids and ceramides are potent activators of ER stress in vitro in hepatocytes [100]. The effects of ceramides on palmitate-induced ER stress depend on their acyl chain lengths. Indeed, whereas treatment with long-chain ceramide exacerbates palmitate-induced ER stress, the very long-chain ceramide species are rather protective [93].

The mechanisms by which ceramide accumulation leads to ER stress activation are not fully understood. It has been shown that ceramide generated by ASMase can trigger ER stress by disrupting calcium homeostasis [101]. Whether ceramide impacts the ER membrane lipid composition which is determinant in ER stress activation [96] is also a possibility that remains to be explored.

Under persistent ER stress, the UPR has been proposed to participate in the progression of steatosis by driving key features of NASH such as inflammation and hepatocyte cell death. IRE1 activates IkB and JNKs, which are implicated in the transcriptional activation of pro-inflammatory cytokines and pro-apoptotic pathways [102]. The PERK branch also activates NF-kB activity. ER stress also contributes to liver inflammation by inducing the NOD-like receptor family pyrin domain containing 3 (NLRP3) inflammasome [103]. It was recently shown that IRE1 upregulates ceramide biosynthesis leading to the release by hepatocytes of ceramide-enriched extracellular vesicles (EVs). These IRE1 stimulated-EVs attract pro-inflammatory monocytes into the liver, triggering inflammation. Genetic or pharmacological inhibition of IRE1 reduces EVs release and inflammation in NASH [104]. Finally, ceramide-induced ER stress triggers apoptotic pathways since the three effectors of the UPR can activate apoptotic proteins such as the PERK-dependent induction of the pro-apoptotic transcription factor C/EBP homologous protein (CHOP) and the IRE1-mediated activation of JNK1 [99]. 

### 4.2. Ceramides and Hepatocyte Cell Death

Rodent as well as clinical studies strongly suggest that hepatocyte cell death is a key event in the progression of chronic liver diseases [105,106]. There is strong evidence that hepatocyte cell death drives inflammation and fibrosis in NASH by promoting the recruitment and activation of immune cells. Although several types of cell death have been described in the physiopathology of NASH, ceramide mainly induces cell death by apoptosis [105,106]. 

Apoptosis is activated by extrinsic and intrinsic pathways that lead to the activation of caspases resulting in proteolysis, nuclear fragmentation, and apoptotic cell death. Treatment with pan-caspase inhibitors or invalidation of specific caspases suppresses apoptosis and improves NASH features in rodents [107,108]. Ceramides have long been known for their pro-apoptotic role. They impact mitochondria driven-apoptosis in several ways. First, they form large channels in membranes of mitochondria increasing the permeability of the mitochondrial outer membrane that leads to the release of cytochrome c, caspase activation, and initiation of apoptosis [109,110]. In parallel, ceramides increase the Bax/Bcl-2 ratio, followed by cytochrome c release, and induce the generation of mitochondrial ROS [111]. The voltage-dependent anion channel 2 was also identified as a direct and specific effector of ceramide-mediated cell death [112].

Blockade of ceramide synthesis was shown to inhibit apoptosis induced by saturated fatty acids and pro-inflammatory cytokines. Liver apoptosis is markedly improved in HFD-fed rats treated with myriocin, the inhibitor of SPT [39]. Acid sphingomyelinase (aSMase) was shown to be a key enzyme for mediating TNFα-induced apoptosis in liver injury. Indeed, the signaling cascade of the proinflammatory cytokine TNF α leads to aSMase activation, resulting in ceramide build-up in cells [113]. aSMase-KO mice were resistant to liver damage and hepatocellular apoptosis [114]. Among ceramide species, C16-ceramides appear to be more harmful to induce apoptosis of hepatocytes. Indeed, mice invalidated for the CerS2 isoform of ceramide synthase display very small concentrations of long-chain (C22–24)-ceramides and high levels of C16-ceramides and develop early hepatocyte apoptosis [61].

### 4.3. Ceramides and Inflammation

Ceramides and inflammation display reciprocal interactions. TNFα stimulates the neutral sphingomyelinase in human hepatocytes inducing an increased ceramide concentration [115]. Another action of inflammation on ceramides occurs through the toll-like receptor 4 (TLR4), a member of the toll-like receptor family that links lipotoxicity to inflammation. TLR4 is expressed in liver cell types, including hepatocytes, monocytes, Kupffer cells, and hepatic stellate cells. Its activation by lipopolysaccharide, a bacterial membrane component, or by saturated fatty acids, particularly palmitic acid, leads to the production of pro-inflammatory cytokines such as TNFα, IL6, and IL1ß. Summers and Scherer establish that TLR4 activation induces ceramide accumulation by increasing the expression of several enzymes involved in de novo ceramide synthesis [116]. In contrast, TLR-4 invalidation reduces ceramide synthesis in the liver of mice fed a HFD. 

Ceramides have been shown to promote inflammation through their interaction with well-known inflammatory targets such as the NLRP3 inflammasome. NLRP3 is a multiprotein complex that mediates caspase-1 activation and secretion of proinflammatory cytokines IL-1β/IL-18 in response to microbial infection and cellular damage. In macrophages, ceramides activate the NLRP3 inflammasome, promoting the cleavage of caspase-1 and the subsequent stimulation of cytokine secretion [117]. 

Adiponectin is an adipokine secreted by adipose tissue that exhibits insulin-sensitizing, anti-inflammatory, and anti-apoptotic functions and the liver is one of its targets [118,119]. Plasma adiponectin concentration is reduced in case of an excess fat mass as observed usually in conditions of NAFLD. It has been proposed that part of the anti-inflammatory effects of adiponectin could be relayed by a decrease in ceramide content. The effects of adiponectin are mediated by two integral membrane receptors named AdipoR1 and AdipoR2. Holland and colleagues proposed that activation of these receptors by adiponectin in cell extracts lowered ceramide levels by activating a ceramidase activity [120]. It was later shown that both receptors possess intrinsic basal ceramidase activity that is enhanced by adiponectin binding [121]. Thus, a reduction of adiponectin plasma concentration in case of excessive fat storage can participate in the inflammation process in the liver. 

### 4.4. Ceramides and Hepatic Fibrosis

Hepatic fibrosis is the consequence of repetitive liver injuries resulting in an excessive accumulation of extracellular matrix (ECM). The cells playing a major role in liver fibrogenesis are HSC. In a healthy liver, HSC are characterized by the presence of numerous lipid droplets enriched in retinyl esters and triglycerides. Following inflammation or other liver injuries, these cells transdifferentiate into myofibroblasts capable of secreting components of the ECM such as type I and type III collagen and fibronectin [122]. Several studies reported an improvement of hepatic fibrosis after inhibition of ceramide biosynthesis by myriocin [21,39]. However, it is difficult to discern from these studies whether these protective effects impact directly on the production of ECM by HSC or indirectly due to the improvement of steatosis and inflammation.

Accumulation of specific ceramide species (C16:0, C22:0 C24:0, C24:1) as well as increased expression of enzymes involved in their synthesis such as CerS2 (synthesis of C22-C24-ceramides), CerS5 (C16-ceramides) are observed in HSC undergoing activation in myofibroblasts in vitro [123]. Induction of aSMase activity and expression is also observed during the transdifferentiation of HSCs into myofibroblasts and this could also participate in the accumulation of ceramide species [124]. Whether this ceramide accumulation upon HSC activation is protective or deleterious remains controversial. Previous studies have reported that ceramide decreases the expression of collagen α1 (the most accumulated collagen species in fibrotic livers) in HSC [125]. It was further shown that these sphingolipid species modulate the transforming growth factor ß (TGF-β) signaling pathway in a concentration-dependent manner, with the highest concentrations being inhibitor and the lowest stimulating [126]. Studies performed in other tissues indicate that ceramides can activate by a “regulated intramembrane proteolysis” process the cleavage of an ER-resident protein named cAMP-responsive element binding protein 3 like 1 (CREB3L1) that in the nucleus binds to Smad4 to transactivate the expression of collagens [127,128]. Whether a similar mechanism occurs in HSCs to promote ECM synthesis is currently unknown. 

The role of ceramides is even less clear when exploring mice models invalidated for enzymes of the sphingolipid metabolism. Indeed, mice heterozygous for aSMase or treated with an aSMase inhibitor are resistant to hepatic fibrosis induced by bile duct ligation or carbon tetrachloride (CCl4) [124,129]. In these studies, cathepsins B and D were identified as targets of ceramide generated by ASMase. Both cathepsins are necessary for HSC transdifferentiation into myofibroblasts. Their inhibition decreased HSC proliferation, the expression of alpha-smooth muscle actin, and other typical markers of HSC activation [130]. On the other hand, ceramide was identified as a powerful inhibitor of the YAP/TAZ (Hippo-related transcriptional coactivators Yes-associated protein/transcriptional coactivator with PDZ-binding motif) mechano-signaling that promotes hepatic fibrosis [131]. Pharmacological inhibition of acid ceramidase (aCDase) by the B13 compound promotes YAP degradation, increases hepatic ceramide content, and decreases fibrosis development in CCl4 treated mice. Genetic invalidation of aCDase in HSC reduces hepatic fibrosis induced by a choline-deficient, l-amino acid–defined, HFD without modification of steatosis and inflammation [132]. It is thus difficult to reconcile the results of these studies since genetic invalidations both leading to an accumulation of ceramides lead to opposite effects on HSC transdifferentiation. Further investigations to discriminate the nature of the ceramide species and their subcellular distribution will be needed to establish if ceramides are protective or harmful in HSC.

**Figure 3:**
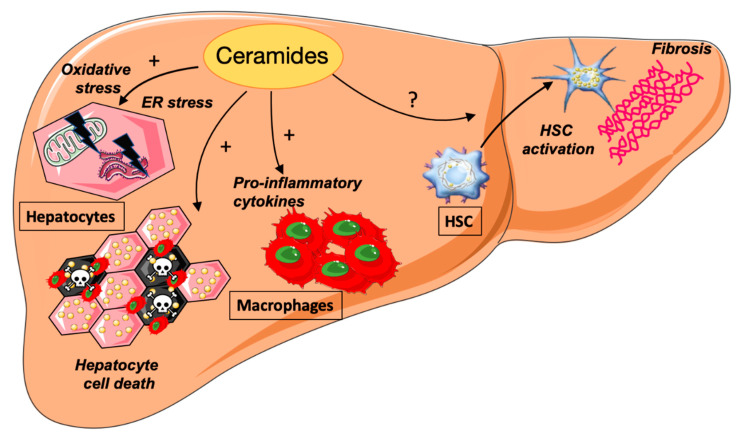
Actions of ceramides on non-alcoholic steatohepatitis (NASH) progression, HSC, hepatic stellate cells.

The actions of ceramides on NASH progression are summarized in Figure 3.

## 5. Ceramides as a Therapeutic Target

Most of the effects of ceramides described here are promoting NAFLD. Therapeutic approaches that target ceramide production could thus be of interest. However, a number of problems must be addressed beforehand. Since ceramides and their derivatives are major components of cellular membranes, it is difficult to consider a total inhibition of ceramide synthesis. Considering the fact that specific ceramide species (e.g., Cer 16:0) are more involved than others in NAFLD onset and progression, one possibility would be to target these species by inhibiting the CerS involved in their synthesis. Some CerS inhibitors do exist already, but none of them target specific CerS with a high degree of selectivity. Fumonisin B1 (FB1), a mycotoxin produced by *Fusarium moniliforme*, remains the best characterized CerS Inhibitor. FB1 acts through competitive-like inhibition towards sphinganine [133]. However, the use of FB1 remains limited due to its toxicity that can induce hepatocarcinogenesis [134]. Additional CerS inhibitors have also been discovered, such as FTY720, an FDA-approved drug for treatment of multiple sclerosis [135] but again, with no specificity towards the CerS. 

Another possibility to reduce ceramide production would be to inhibit DES1 activity/expression [136]. Indeed, fenretinide, a drug that inhibits Des1 activity and blocks ceramide biosynthesis, prevents hepatic steatosis in animal models [137]. 

More comprehensive knowledge of the ceramide interaction with their targets and the reasons why specific ceramide species are deleterious where others are rather protective is certainly a prerequisite for the development of adequate drugs. In addition, one must keep in mind that ceramides promote apoptosis and their use is considered for cancer treatment. Ceramide content was reported to be reduced in HCC, leading to a decrease in apoptosis [138]. Several drugs such as vinblastine and sorafenib activate CerS and increase ceramide content inside the liver, resulting in a powerful antitumor effect [139,140]. In addition, C6-ceramide has been shown to increase the anti-tumor immune response and to slow the growth of liver tumors in mice [141]. These data show that hepatic accumulation of ceramides can induce the appearance of NASH but on the other hand, their absence can promote HCC. Thus, modulating ceramide concentration one way or another must be carefully controlled.
